# Tackling tumor microenvironment through epigenetic tools to improve cancer immunotherapy

**DOI:** 10.1186/s13148-021-01046-0

**Published:** 2021-03-24

**Authors:** Iris Lodewijk, Sandra P. Nunes, Rui Henrique, Carmen Jerónimo, Marta Dueñas, Jesús M. Paramio

**Affiliations:** 1 Molecular Oncology Unit, Centro de Investigaciones Energéticas, Medioambientales Y Tecnológicas (CIEMAT), 28040 Madrid, Spain; 2 Biomedical Research Institute I+12, University Hospital “12 de Octubre”, 28041 Madrid, Spain; 3 Cancer Biology and Epigenetics Group – Research Center, Portuguese Oncology Institute of Porto (CI-IPOP), 4200-072 Porto, Portugal; 4 Department of Pathology, Portuguese Oncology Institute of Porto, 4200-072 Porto, Portugal; 5 Department of Pathology and Molecular Immunology, Institute of Biomedical Sciences Abel Salazar – University of Porto (ICBAS-UP), 4050-313 Porto, Portugal; 6 Centro de Investigación Biomédica en Red de Cáncer (CIBERONC), 28029 Madrid, Spain

**Keywords:** Epigenetics, Immunotherapy, Tumor microenvironment, Therapy, Bladder cancer

## Abstract

**Background:**

Epigenetic alterations are known contributors to cancer development and aggressiveness. Additional to alterations in cancer cells, aberrant epigenetic marks are present in cells of the tumor microenvironment, including lymphocytes and tumor-associated macrophages, which are often overlooked but known to be a contributing factor to a favorable environment for tumor growth. Therefore, the main aim of this review is to give an overview of the epigenetic alterations affecting immune cells in the tumor microenvironment to provoke an immunosuppressive function and contribute to cancer development. Moreover, immunotherapy is briefly discussed in the context of epigenetics, describing both its combination with epigenetic drugs and the need for epigenetic biomarkers to predict response to immune checkpoint blockage.

**Main body:**

Combining both topics, epigenetic machinery plays a central role in generating an immunosuppressive environment for cancer growth, which creates a barrier for immunotherapy to be successful. Furthermore, epigenetic-directed compounds may not only affect cancer cells but also immune cells in the tumor microenvironment, which could be beneficial for the clinical response to immunotherapy.

**Conclusion:**

Thus, modulating epigenetics in combination with immunotherapy might be a promising therapeutic option to improve the success of this therapy. Further studies are necessary to (1) understand in depth the impact of the epigenetic machinery in the tumor microenvironment; (2) how the epigenetic machinery can be modulated according to tumor type to increase response to immunotherapy and (3) find reliable biomarkers for a better selection of patients eligible to immunotherapy.

**Supplementary Information:**

The online version contains supplementary material available at 10.1186/s13148-021-01046-0.

## Background

The epigenome is defined by heritable alterations in gene expression, either activation or suppression, without altering the DNA nucleotide sequence. The mechanisms responsible for these changes can be broadly divided into altered gene accessibility for the transcriptional machinery, disrupted chromatin organization or modulation of gene expression at the post-transcriptional level through altered mRNA translation mainly by non-coding RNAs, including miRNAs. Fundamental mechanisms for epigenetic regulation include DNA methylation, histone modifications, chromatin remodeling and non-coding RNA interference [1]. Nevertheless, these modifications of the RNA sequence and their associated regulatory factors represent functionally relevant changes to the transcriptome without altering the RNA ribonucleotide sequence, recently encompassed by the term ‘epitranscriptomics’ [2]. Since epitranscriptomics is recognized as a different area of study that goes beyond the scope of this review, we will only focus on DNA methylation, histone modifications and chromatin remodeling as epigenetic regulatory mechanisms. Those mechanisms are briefly discussed below.

Epigenome functions are essential for normal gene expression, and their modifications affect primary cellular processes like proliferation, differentiation, and apoptosis. Even though its effect on human carcinogenesis is not entirely acknowledged, epigenetic dysfunction is a rising hallmark of malignancy. As epigenetic modifications are essential in the regulation of normal gene expression, epigenetic deregulation results in aberrant gene expression patterns which have been found to favor tumorigenesis, among others [1].

Whereas the role of epigenetic modifications in cancer research has been mainly focused on cancer cells, rising evidence indicates their contribution to the development of a favorable tumor microenvironment (TME), including their effect on surrounding cell phenotypes like fibroblasts, immune cells, endothelial and inflammatory cells, blood and lymphatic vascular networks, and the extracellular matrix (Table 1) [3]. Nevertheless, due to the extreme complexity of the variety of cells and their potential epigenetic modifications affecting tumorigenesis, this review will be focused on the epigenetic regulation of different immune cell types in the TME and their involvement in the generation of a cancer-prone TME.

**Table 1:** Cell populations contributing to the development of a favorable tumor microenvironment

TME components	Reported in various tumor types, including:	Characteristics controlled by epigenetic regulation	Contribution to favorable TME		References
Pro-tumor DCs	Bladder, Breast, Colon, Colorectal, Esophageal, Gastric, Glioma, Intestinal, Liver, Lung, Lymphomas, Osteosarcoma, Ovarian, Pancreas, Prostate	Enhanced production of pro-tumorigenic cytokine IL-6 Augmented secretion of immunosuppressive Galectin-1	Tumor-infiltrating DCs show an immune-tolerant phenotype favoring tumor growth		[4–21]
Immunosuppressive MDSCs	Bladder, Breast, Colorectal, Head and Neck, Liver, Lung, Pancreas, Prostate, Renal	Expression of immunosuppressive factors S100A8, S100A9 and Arg1	MDSCs promote immune suppression favoring tumor growth and metastasis		[16, 22–31]
TAMs	Bladder, Breast, Colon, Cervical, Gastric, Glioma, Lung, Lymphoma, Melanoma, Neuroblastoma, Ovarian, Pancreas, Prostate	Increased expression of Arg1 Activation of ALOX15 Enhanced expression of PPARγ Decreased NFkB signaling and suppression of IL-1β expression	Immunosuppressive subsets of TAMs promote tumor growth		[16, 32–42]
TILs	Bladder, Brain, Breast, Colorectal, Gastric, Genitourinary, Glioblastoma, Glioma, Head and Neck, Liver, Lung, Melanoma, Meningioma, Ovarian, Pancreatic, Prostate, Renal	*Impaired TILs CD8* + *infiltration* Downregulation of CXCL9 and CXCL10 chemokine expression Elevated levels of PD-1 *Exhausted CD8* + *T cells* Enhanced expression of CTLA4, PDCD1 and LAG3	Impaired CD8 + T cell functions as well as T-cell exhaustion seem to play a major role in the generation of an immunosuppressive TME		[43–46]
Mature Tregs	Bladder, Breast, Colorectal, Glioblastoma, Lung, Lymphoma, Melanoma, Ovarian, Pancreas	Downregulation of genes involved in effector T-cell activation	Mature Tregs provide a tumor-supportive microenvironment		[16, 47–50]
NK cells	Breast, Colorectal, Gastric, Melanoma, Multiple myeloma, Pancreas, Renal	Decreased expression of activating NKG2D NK-cell receptor	Impaired NK-cell mediated anti-tumor immune response		[51–54]
Fibroblasts			Fibroblasts in the TME differentiate into cancer-associated fibroblasts (CAFs), representing one of the main components in the tumor stroma. The majority of the studies show pro-tumoral functions for CAFs, which include immune suppression, extracellular remodeling and angiogenesis		[55–57]
Endothelial cells			Angiogenesis refers to the de novo formation of blood vessels, a process essential for tumor growth. This blood vasculature consists of tumor endothelial cells (TECs), which line the insides of the blood vessels. Different characteristics between TECs and normal endothelium have been described		[58, 59]
Blood and lymphatic vascular networks			Blood and lymphatic vascular networks play a key role in supporting tumor growth and progression. They support oxygen supply and adequate nourishment to the tumor and provide a gateway for metastasizing tumor cells		[60]
Extracellular matrix			The tumor extracellular matrix (ECM) differs greatly from the ECM in normal tissue, not only in amount of deposition but also in organization and composition. The tumor ECM is generally stiffer, a characteristic that has been associated with an increase in nuclear localization of transcription factors able to drive epithelial-mesenchymal-transition and, consequently, tumor metastasis		[61]

We further discuss the inhibition of epigenetic modulators as therapeutic option to modify the immunosuppressive TME, and we provide an overview on immunotherapy and the potential of epigenetic biomarkers of response to this therapy. Finally, the application and success of immunotherapy as well as the inhibition of epigenetic processes involved in immune activation will be briefly discussed in the context of bladder cancer (BC).

## Epigenetic regulatory mechanisms

### DNA methylation

**Fig. 1:**
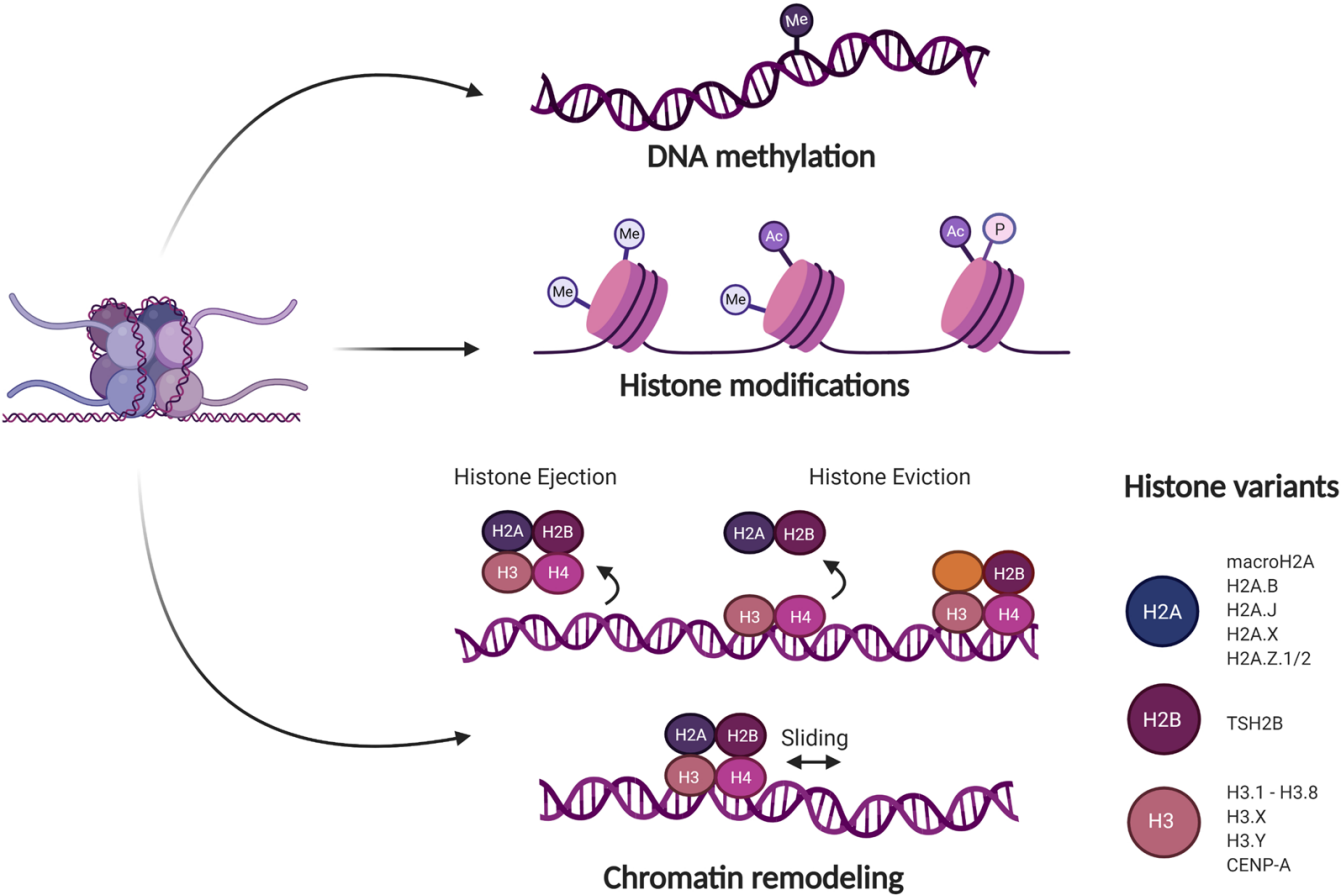
DNA methylation, histone modifications and chromatin remodeling as regulatory mechanisms of epigenetic gene regulation. DNA methylation represents a process by which methyl groups are transferred onto the 5′ position of a cytosine molecule, commonly in the context of CpG sites, without altering the DNA sequence. Histone modifications include post-translational modifications at the histone N-terminal tails, such as acetylation, methylation and phosphorylation, causing chromatin structure alterations. Changes in nucleosome position are also responsible for rearrangement of chromatin structure, a process known as chromatin remodeling. Nucleosomes can be affected in several ways, including nucleosome sliding, nucleosome ejection and histone eviction. Histone variants add further complexity to epigenetic regulation of the genome. Various histone variants are characterized for H2A, H2B and H3. All these mechanisms are highly interrelated and play an important role in the regulation of gene accessibility to the transcriptional machinery

DNA methylation represents a process by which methyl groups are transferred onto the 5′ position of a cytosine molecule without altering the DNA sequence. Methylation commonly occurs on the cytosine of CpG sites, meaning that the cytosine molecule precedes a guanine. DNA regions with a higher density of these CpG sites (so-called CpG islands) have been found throughout the genome, mostly coinciding with gene regulatory regions. This way, methylation of CpG islands plays an important role in the regulation of normal gene expression (Fig. 1) [62, 63].

DNA methyltransferases (DNMTs) are the enzymes responsible for the control of DNA methylation patterns through maintenance DNA methylation and de novo DNA methylation. In general, DNMT1 is the enzyme responsible for maintenance of inherited DNA methylation, whereas DNMT3a and DNMT3b provide de novo DNA methylation. Nevertheless, DNMT3a and DNMT3b methyltransferases have been described to perform maintenance methylation as well, and DNMT1 has also been found to carry out de novo DNA methylation [63].

Alterations in DNA methylation status have been described in various diseases, including cancer. For example, gene silencing of tumor suppressor genes (TSGs) is the result of the hypermethylation of CpG islands in the promoter regions of those genes. TSGs are mainly involved in biological pathways like cell cycle control, DNA repair and apoptosis, and its silencing has been frequently found in tumorigenesis [64].

### Histone modifications

Chromatin structure changes as a result of dynamic processes involving post-translational modifications (PTMs) at the histone N-terminal tails. Various PTMs can be distinguished, including histone acetylation, methylation and phosphorylation as well as less known ubiquitylation, deamination and sumoylation, which affect chromatin packaging and availability to the gene transcription machinery (Fig. 1) [65]. Currently known histone PTMs have been extensively reviewed. Here, we briefly mention the three most common histone PTM activities.

Histone acetylation consists of the reversible addition of acetyl groups to the histone tail by histone acetyltransferases (HATs), which weakens the DNA-histone bonds and allows binding of transcription factors. Contrarily, histone deacetylases (HDACs) remove those acetyl groups, allowing for compact wrapping of the DNA around histones, disabling the access of other enzymes. Regarding histone methylation, the transfer of methyl groups is a reversible process regulated by histone methyltransferases and demethylases. The attachment of a methyl group to the histone tail may differentially affect gene expression depending on the specific residue modified. Likewise, the interaction between the DNA and histone tails is regulated by histone (de)phosphorylation processes [66].

### Chromatin remodeling

Changes in nucleosome position have also been found responsible for rearrangement of chromatin structure, a process known as chromatin remodeling. Nucleosomes, consisting of a histone core (H2A, H2B, H3 and H4) wrapped by an approximately 150-bp DNA sequence, can be affected in several ways, including nucleosome sliding, nucleosome ejection and histone eviction. Nucleosome sliding represents the movement of the histone octamer across the DNA sequence, whereas nucleosome ejection implies the complete segregation of the histone core from the DNA. Histone eviction includes the disintegration of the core histone octamer trough removal or replacement of H2A–H2B dimers (Fig. 1) [67].

Since nucleosome sliding and ejection as well as removal of the H2A–H2B dimers result in DNA exposition and nucleosome destabilization, these processes play an important role in the regulation of gene accessibility to the transcriptional machinery.

### Histone variants

Histone variants add further complexity to epigenetic regulation of the genome. They represent a unique protein sequence compared to core histones and can be identified by a combination of variant-specific proteins and chromatin remodeling complexes which control their localization within the genome. Various histone variants are characterized for H2A (such as macroH2A, H2A.B, H2A.J, H2A.X and H2A.Z.1/2), H2B (including TSH2B) and H3 (like H3.1 till H3.8, H3.X, H3.Y and CENP-A), whereas no variants have been described for H4. Additionally, histone variants are subjected to post-translational modifications which elaborates the epigenetic control of gene expression (Fig. 1) [68, 69].

## Epigenetic regulation of immune cell function in TME

A favorable TME is characterized by immune tolerance. Cancer cells employ a variety of epigenetic regulated-immune escape mechanisms, including downregulation of tumor-associated antigens (TAAs), loss of antigen processing and presentation machinery (APM) as well as expression of a tumor-promoting balance in co-stimulatory and co-inhibitory molecules (also known as immune checkpoint receptors). Whereas these processes have been extensively studied and reviewed, epigenetic alterations affecting immune cell function in the TME represent a growing area of investigation. The epigenetic-induced immunosuppressive function of dendritic cells (DCs), myeloid-derived suppressor cells (MDSCs), tumor-associated macrophages (TAMs), tumor-infiltrating lymphocytes (TILs), regulatory T cells (Tregs) and natural killer cells (NK cells) in the TME will be discussed below.

### Dendritic cells

DCs represent important antigen-presenting cells (APCs) that mediate antigen-specific anti-tumor immune responses mainly through the activation of T cells. Whereas these cells are normally able to capture TAAs expressed on cancer cells through accurate MHC expression and cross-present them to cytotoxic T cells (CTLs) by the expression of co-stimulatory molecules, resulting in tumor elimination, tumor-infiltrating DCs show an immune-tolerant phenotype favoring tumor growth [18]. Next to low MHC expression and repression of various co-stimulatory molecules in tumor-infiltrating DCs, epigenetic alterations affecting DC polarization and activity are suggested to impair an effective anti-tumor immune response.

**Fig. 2:**
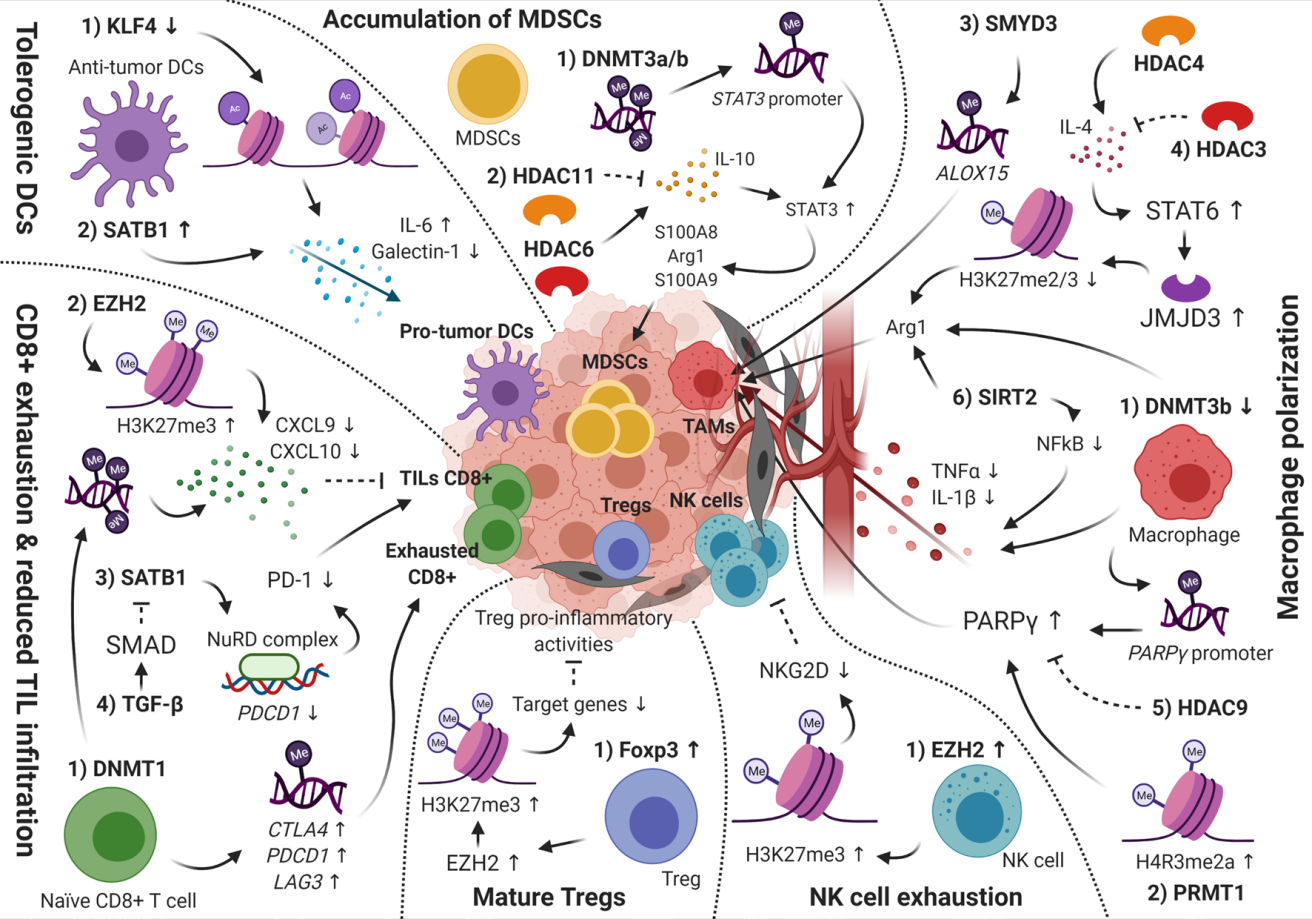
Epigenetic regulation of immune cells in the tumor microenvironment. Decreased KLF4 and increased SATB1 expression affect IL-6 (upregulation) and Galectin (downregulation) expression, remodeling anti-tumor DCs into pro-tumor DCs. MDSCs expansion, accumulation and recruitment are favored by STAT3-induced expression of immunosuppressive factors S100A8, Arg1 and S100A9. In this cell population, STAT3 expression is controlled by DNMTT3a/b, HDAC6 and HDAC11. Macrophages can convert into TAMs under the influence of multiple epigenetic factors, including DNMT3b, PRMT1, HDAC3/4, HDAC9 and SIRT2, favoring acquisition of the M2 phenotype through various pathways, such as increased PPARγ and Arg1 expression as well as downregulation of inflammatory factors TNF-α and IL-1β. SMYD3 activates M2 marker ALOX15. Impaired NK-cell anti-tumor cytotoxicity can be the result of increased EZH2 expression, which downregulates activating NK-cell receptor NKG2D through enhanced H3K27me3 levels. The same way, EZH2 also regulates inhibition of regulatory T-cell pro-inflammatory activities. Naïve CD8 + T-cells differentiate into TILs or exhausted CD8 + cells dependent on epigenetic profile. Whereas specific DNA methylation patterns of *CTLA4*, *PDCD1* and *LAG3* are identified in exhausted CD8 + T-cells, DNMT1 and EZH2 inhibit CD8 + TILs infiltration through downregulation of CXCL9 and CXCL10 chemokines. TGF-β and SATB1 affect TILs infiltration by controlling PD-1 expression. DCs, dendritic cells; MDSCs, myeloid-derived suppressor cells; TAMs, tumor-associated macrophages; NK, natural killer; Tregs, regulatory T-cells; TILs, tumor-infiltrating lymphocytes

For example, dynamic changes in the levels of chromatin regulator ‘special AT-rich sequence binding 1 (SATB1)’ are essential for the generation of inflammatory DCs and their anti-tumorigenic activity. SATB1 recruits chromatin remodeling complexes to anchored DNA regions, consisting of a characteristic ‘ATC-sequence context’ (well-mixed A’s, T’s and C’s but not G’s on a single strand), thereby controlling gene transcription over long-distance DNA sequences through the regulation of nucleosomal positioning and histone modification [70]. Additionally, the recruitment of HATs and HDACs directly to gene promoter regions has been reported [71]. A continuous increased expression of SATB1 has been described to convert inflammatory anti-tumor DCs into pro-tumor DCs by enhanced secretion of pro-tumorigenic cytokine IL-6 and immunosuppressive factor Galectin-1, activating immune-evasive pathways in these cells [7]. Accordingly, SATB1 has been found to be overexpressed in a wide range of tumors, including breast, lung, pancreas, colorectal, liver, bladder, prostate and ovarian cancer, and has been associated with tumor progression and poor prognosis [19]. Additionally, next to its role in the direct activation of *IL-6* transcription, Kruppel-like factor 4 (KLF4) modulates IL-6 production at the post-translational level through histone acetylation. Decreased expression of KLF4 has been described in many tumors, including esophageal, lung, gastric, intestinal, colon and prostate cancer, leading to altered production of cytokine IL-6 in DCs (Fig. 2, Table 1) [20, 21].

Taken together, increasing our knowledge on tumor-induced epigenetic modifications affecting DC polarization and activity might help modifying the TME to become more “receptive” to the development of an effective anti-tumor response.

### Myeloid-derived suppressor cells

MDSCs represent immature myeloid cells and are mainly characterized by their immunosuppressive function providing tumor immune evasion [27]. These cells are known to have a major impact on cancer progression as the TME has been found to support this cell population, promoting MDSC persistence, proliferation and function. Indeed, the presence of MDSCs has been associated with poor prognosis and reduced patients’ survival in many cancer types, including head and neck, breast, lung, kidney and prostate [26, 27]. Several studies have suggested the role of epigenetic mechanisms in MDSC accumulation and functions.

For example, an elevated expression of signal transducer and activator of transcription 3 (STAT3) has been defined in several tumor types including lung, pancreas and renal cancer [28–31]. Overexpression of STAT3 can be the result of promoter silencing of DNMT3a and DNMT3b through hypermethylation, followed by promoter hypomethylation of the *STAT3* gene. Besides, Villagra et al. proposed HDAC11 as a transcriptional repressor of IL-10 (a STAT3-activating cytokine) through interaction with the IL-10 promoter at the chromatin level and indicated that elevated levels of STAT3 in APCs might be associated with the absence of HDAC11 [72]. More recently, HDAC11 has been described as an essential regulator of IL-10 levels in myeloid cells and its role in the MDSC expansion was demonstrated [73]. Moreover, Cheng et al. showed that HDAC6 has a regulatory function in STAT3 activation in the MDSC population. Surprisingly, HDAC6 seems to act as a transcriptional activator of *IL-10* expression [74]. Next to their possible individual implications, HDAC6 and HDAC11 have been reported to interact and be recruited together toward the *IL-10* promoter site where they control *IL-10* transcription and subsequent STAT3 expression [74]. Increased STAT3 expression leads to augmented expression of immunosuppressive factors S100A8 and Arginase 1 (Arg1) in MDSCs. Additionally, the induction of these proteins, together with the STAT3-mediated induction of S100A9 expression, has been shown to provide expansion, accumulation and recruitment of immunosuppressive MDSCs in TME (Fig. 2, Table 1) [29, 75].

As abovementioned, this MDSC population represents immature myeloid cells which fail to differentiate into macrophages and DCs. Several studies already described the accumulation of immature myeloid cells as a result of retinoblastoma gene (*Rb*) transcriptional silencing [76, 77]. Concordantly, Young et al. proposed that HDAC2 might be the epigenetic regulator provoking Rb transcriptional silencing in this cell population through its direct interaction with the *Rb1* gene promoter [78].

Taken together, the MDSC population represents a major barrier for immunotherapy. Accordingly, further research is needed to increase our knowledge on MDSCs in the TME and be able to improve the ability to revert their immunosuppressive function.

### Tumor-associated macrophages

TAMs represent the main component of the immune infiltrates in TME of solid tumors and have frequently been associated with worse prognosis [79]. By continuously sensing their surrounding environment, this cell population has refined regulatory epigenetic mechanisms to manage their polarization state. Depending on their polarization into classically activated (M1) or alternatively activated (M2) macrophages, they inhibit or promote tumor growth, respectively. Epigenetic modifications have been widely shown to be involved in macrophage differentiation, activation and survival [80].

Yang et al. indicated a significant role for DNMT3b in macrophage polarization. They showed that DNMT3b knockdown induces elevated expression of M2 macrophage markers, such as Arg1, as well as increased Arg1 function. In concordance with these results, Arg1 activity has been reported to define immunosuppressive subsets of TAMs. Additionally, DNMT3 knockdown resulted in significantly decreased expression of inflammatory genes, such as *TNF-α* and *IL-1β*, emphasizing the importance of DNMT3b in the regulation of both macrophage differentiation and inflammation [81, 82]. Moreover, DNMT3b has been reported to methylate the peroxisome proliferator-activated receptor γ (PPARγ) promoter region.

Protein arginine methyltransferase 1 (PRMT1) has been reported as a positive regulator of PPARγ-dependent M2 polarization through methylation of the arginine located at residue 3 on the tail of histone 4 (H4R3me2a). Furthermore, Ishii et al. demonstrated that expression of M2 macrophage markers seems to be epigenetically controlled by convertible changes in H3K4 and H3K27 methylation [83]. Accordingly, H3K4 methyltransferase SET and MYND Domain 3 (SMYD3) has been shown to play a role in M2 differentiation. Kittan et al. [84] showed that the increased expression of SMYD3 is associated with the methylation and activation of the M2 marker arachidonate 15-lipoxygenase (ALOX15). The only histone demethylase recognized as a crucial regulator of M2 polarization is Jumonji domain-containing protein D3 (JMJD3), a H3K27 demethylase. IL-4-induced STAT6 activation leads to STAT6-mediated increased expression of JMJD3, provoking H3K27me2/3 demethylation and subsequent transcriptional activation of several M2 marker genes, including *Arg1* [83, 85]. IL-4 increased expression has been found in various tumor types, including breast, lung, pancreatic, colon, bladder and ovarian carcinomas [42].

Whereas the role of HATs in macrophage polarization remains unclear, the function of HDACs as epigenetic modifiers in the regulation of M2 differentiation and phenotypic control has been explored by various studies. Mullican et al. indicated that HDAC3 activity leads to suppressed IL-4 activity through deacetylation of histone tails at regulatory sequences. Together with the finding that HDAC3 knockdown resulted in decreased inflammatory gene expression, HDAC3 has been proposed to negatively regulate M2 polarization [86, 87]. HDAC9 has also been described as a negative regulator of M2 phenotype as HDAC9 deficiency results in PPARγ promoter acetylation and increased PPARγ expression levels, promoting M2 polarization and downregulating M1 phenotype inflammatory genes [88]. Contrarily, HDAC4 positively regulates the M2 phenotype through IL-4-activated HDAC4-induced STAT6 signaling and Arg1 expression [89]. Another HDAC, SIRT2, positively controls M2 polarization through its function in the expression of M2 macrophage markers, such as Arg1, and downregulation of M1 polarization by NFkB acetylation, provoking decreased NFkB signaling and suppression of IL-1β expression (Fig. 2, Table 1) [90].

Accordingly, targeting these epigenetic enzymes responsible for polarization of TAMs into M2 macrophages would prevent their tumor-supporting function. Nevertheless, it should be considered that these regulators disclose secondary functions and that histone-modifying enzymes also affect proteins other than histones.

### Tumor-infiltrating lymphocytes

TILs represent the major component of the adaptive immune system in the TME and can be classified into two main categories: 1) CD4-expressing T cells (CD4 + T cells), which can differentiate into the T helper1 (Th1) or T helper2 (Th2) phenotype, and 2) CD8-expressing T cells (CD8 + T cells), which are able to eliminate tumor cells after differentiation into cytotoxic effector T lymphocytes.

Whereas the important role of CD8 + CTLs in anti-tumor immune response has been known for many years, the potential importance of CD4 + Th cells in the generation and maintenance of anti-tumor activity has only recently been reported [91]. Even though further research is needed to find out whether and, if so, how epigenetic mechanisms affect CD4 + cells in an immunosuppressive TME, DNA demethylation has been reported to play an important role in differentiation of CD4 + T cells toward Th1/Th2 lymphocytes [92].

Additionally, epigenetic modulation has been found to control rapid activation and differentiation of naïve CD8 + into CTLs upon antigen stimulation. For example, Peng et al. associated DNMT1-mediated DNA methylation and enhancer of zeste homolog 2 (EZH2)-mediated H3K27 trimethylation with impaired T-cell infiltration in the TME through downregulation of CXCL9 and CXCL10 chemokine expression [93]. Furthermore, Yang et al. reported that whole-genome methylation profiling showed a distinct methylome pattern for tumor-reactive CD8 + T cells compared to the naïve subtype. Moreover, specific DNA methylation patterns have been discovered in exhausted CD8 + T cells. *PDCD1* and *CTLA4* expression in exhausted CD8 + T cells has been found to be epigenetically controlled by DNA demethylation, and the *LAG3* gene has been found methylated in naïve cells but demethylated during the activation of naïve CD8 + T cells [94]. Ghoneim et al. [95] found that high programmed death 1 (PD-1)-expressing tumor-infiltrating CD8 + T cells in prostate cancer display exhaustion-associated DNA methylation patterns. Stephen et al. further showed that chromatin organizer Satb1 recruits the nucleosome remodeling deacetylase complex to regulatory regions of the *Pdcd1* gene, reducing PD-1 expression levels upon T-cell activation. Nevertheless, Satb1 is known to be downregulated by Smad proteins under the influence of TGF-β, an immunosuppressive cytokine found to play a relevant role in cancer, resulting in elevated PD-1 levels. Accordingly, Satb1 prevents premature T-cell exhaustion by controlling PD-1 expression, a pathway that is altered in cancer, causing reduced anti-tumor activity [96, 97]. Another mechanism underlying tumor-specific T-cell dysfunction in tumor progression is represented by chromatin state dynamics. Philip et al. reported that naïve T cells differentiate into a dysfunctional but reprogrammable chromatin state upon tumor antigen recognition in premalignant lesions, which converts into a fixed non-reprogrammable dysfunctional state during tumor progression. The presence of surface markers CD101 and CD38 has been associated with reduced reprogrammability of high PD-1-expressing tumor-infiltrating CD8 + T cells, a finding with important clinical relevance as these markers can be used to discriminate reprogrammable from non-reprogrammable PD-1 high T cells within the heterogeneous TIL populations [98]. This mechanism might explain why certain patients do not respond to therapies based on immune-checkpoint blockade as well as it provides new insights in possible strategies to revert non-reprogrammable PD-1 high T cells into tumor-reactive CD8 + T cells (Fig. 2, Table 1).

Taken together, impaired CD8 + T cell functions seem to play a major role in the generation of an immunosuppressive TME. Importantly, the prevention of T-cell exhaustion might represent a potential strategy to reverse a TIL-mediated immunosuppressive TME.

### Regulatory T cells

Tregs represent a functionally different T cell population which is essential for the maintenance of homeostasis and immune tolerance. Accordingly, mature Tregs provide a tumor-supportive microenvironment [47]. Various studies have reported a key role for Foxp3 in the development of these cells as well as their function, and epigenetic regulation of Tregs through Foxp3 has been emphasized by recent studies [99, 100].

Moreover, epigenetic modifications controlling Treg development and function have been found to play an important role in the establishment of an immunosuppressive TME. Ohkura et al. reported that Treg maturation involves the generation of genome-wide CpG DNA hypomethylation pattern, needed for Treg-specific gene expression and immunosuppressive activity [101]. Besides, Foxp3 seems to exert an EZH2-mediated repressive role upon CD28-mediated Treg activation as target genes show elevated H2K27me3 levels. As CD28 not only provides a key role in the stimulation of Tregs, but also in effector T-cell (CD4+/CD8+) activation, this suppressive role of Foxp3 might be essential to preserve the Treg-specific gene expression profile upon T cell stimulation through downregulation of genes involved in the effector T-cell activation [102]. Indeed, Wang et al. showed that inhibition of EZH2 resulted in Treg-mediated pro-inflammatory activities in the TME, supporting the generation of an effector T-cell-mediated anti-tumor immune response (Fig. 2, Table 1) [103].

Taken together, targeting the maturation of functional Tregs might be a potential strategy to convert an immunosuppressive TME into a microenvironment able to provide anti-tumor activity.

### Natural killer cells

The NK cell population forms part of the innate immune system and is able to control tumor growth by their ability to recognize and eliminate tumor cells. Epigenetic modification has been reported to play an key role in the NK cell maturation, differentiation and activation [104]. Regulation of the effector function of this cell population mainly depends on the balance between inhibiting and activating receptors present on NK cell surface, the activation status of which seems to be epigenetically modulated, as well. Accordingly, rising evidence indicates the involvement of epigenetic processes in impairing NK-cell mediated anti-tumor immune response.

An impaired NK-cell-mediated anti-tumor immune response is highly associated with NK-cell exhaustion because of diminished expression of activating receptors and increased expression of inhibitory receptors, among others. Specific activating NK-cell receptors include NKG2D, NKp30, NKp44, NKp46 and DNAM-1/CD226, whereas inhibitory receptors are represented by PD-1, TIM-3, TIGIT and CD94-NKG2A. Fernandez-Sanchez et al. reported the involvement of DNA methylation and histone acetylation in the regulation of NKG2D levels, with *NKG2D* gene demethylation and H3K9 acetylation providing *NKG2D* expression [105]. Nevertheless, whether reduced expression of this receptor is due to *NKG2D* hypermethylation remains unknown. Additionally, Ogbomo et al. proposed that the regulation of NKp30 and NKp46 expression levels is controlled by histone acetylation [106]. Using an HDAC inhibitor (HDACi), they showed that the suppression of NK-cell activity is caused by decreased expression of NKp30 and NKp46, but is independent of activating NKG2D, NKp44 and DNAM-1 expression levels as well as inhibitory *NKG2A* expression. Finally, Yin et al. revealed that enhanced levels of the activating NKG2D receptor are associated with elevated NK cell expansion and cytotoxicity against the tumor. Inhibition of EZH2 activity has been associated with decreased H3K27me3 levels, providing increased expression of the NKG2D receptor (Fig. 2, Table 1) [107, 108].

Although epigenetic modifications affecting NK cell development and function are widely examined, further studies are needed to increase our knowledge on the epigenetic regulation of NK cells and the potential of epigenetic enzymes/markers as therapeutic targets.

## Inhibition of epigenetic modulators as therapeutic option to modify the immunosuppressive TME

A favorable TME, created by tumor cells affecting different immune cell populations, forms a major barrier for cancer therapy. Nevertheless, the study into epigenetic mechanisms underlying the generation of this immunosuppressive TME currently represents subject of utmost interest. As previously stated, a variety of epigenetic modifications affects the phenotypes of diverse immune cell populations in the TME to become immunosuppressive. Accordingly, modifying the TME to become more “receptive” to the development of an effective anti-tumor response could be achieved by molecular re-wiring using pharmacologic modulators of epigenetic enzymes. In this regard, when considering the use of compounds targeting the epigenetic machinery, it is worth considering that these compounds not only affect tumor cells, but also TME cells. Accordingly, a proper selection of inhibitors could become a two-edge sword to tackle tumors.

For example, SATB1 represents an attractive therapeutic target as it modifies different immune cell populations under tumor's influence, including DCs and CTLs. The statins fluvastatin and simvastatin have been found to suppress SATB1 expression, probably acting at the post-translational level [109, 110]. Additionally, in vivo silencing of *Satb1* expression in tumor-associated DCs was found to diminish immunosuppression in the TME, boost T-cell mediated anti-tumor activity and delay tumor progression [7]. In the context of immunotherapy, Satb1 has been described as a possible TAA that can be recognized by CD8 + T cells. Accordingly, Wang et al. proposed that Satb1-derived epitope might be used as immune target for cancer vaccine generation [111].

Furthermore, the importance of HDAC activation and IL-6 signaling in controlling the immunosuppressive function of MDSCs as well as its recruitment to the TME has been reported by Nair et al. [26]. Various studies demonstrated the potential of entinostat, a class I HDACi, as therapeutic compound to modulate the immunosuppressive TME through inhibition of MDSC activity. Besides, the application of entinostat not only resulted in decreased MDSC function, but also augmented the effect of checkpoint inhibitor treatment [112–115]. Kim et al. reported that entinostat reduces the MDSC population and the combination of entinostat with PD-1 and cytotoxic T-lymphocyte-associated protein 4 (CTLA-4) antibodies resulted in an elimination of approximately 80% of the tumor, whereas the use of immune checkpoint inhibitors against PD-1 and CTLA-4 alone did not provide an anti-tumor immune response [113]. The potential role of HDACi in priming the TME for enhanced response to immunotherapy has been further emphasized by Briere et al., who obtained similar results using a class I/IV HDACi (mocetinostat) in combination with anti-programmed death-ligand 1 (PD-L1) antibody [114]. Additionally, Orillion et al. observed reduced macrophage population in the TME after entinostat treatment [112]. Other potential therapeutic targets to suppress MDSCs function would be HDAC2, HDAC6 and HDAC11. Nevertheless, further studies are needed to investigate the therapeutic potential of their corresponding inhibitors [78, 115, 116].

Regarding the polarization of TAMs into M2 macrophages, targeting the enzymes responsible for the acquisition of M2 phenotype would attenuate their tumor-promoting function. Accordingly, Tikhanovich et al. reported the therapeutic potential of AMI-1, a PRMT1 inhibitor, inhibiting M2 processes [117]. Additionally, GSK-J4 might diminish the immunosuppressive, tumor-supporting function of M2 macrophages through KDM6B (a lysine-specific demethylase that demethylates H3K27me2 or H3K27me3) inhibition. Nevertheless, in concordance with the essential role of KDM6B in both M1 and M2 polarization, GSK-J4 has also been found to inhibit the expression of TNF and other M1 inflammatory cytokines [118, 119]. Noteworthy is the effect of histone-modifying enzymes on proteins other than histones and the importance of non-histone protein modifications in macrophage-polarizing process. Moreover, macrophage polarization is a complex dynamic process in which most epigenetic enzymes are neither involved exclusively in the polarization toward M1 or M2 phenotype nor do they all have opposing roles in M1 versus M2 phenotype acquisition [120]. Accordingly, the discovery of therapeutic epigenetic targets in this cell population is very challenging.

Another attractive therapeutic target is EZH2, responsible for the immunosuppressive phenotype of several immune cell populations in the TME, including TILs, Tregs and NK cells. This epigenetic regulator has already been extensively studied for its potential as therapeutic target to convert the immunosuppressive TME into an immune-promoting microenvironment. Various studies described an enhanced effector-T cell infiltration and cytotoxic activation in the TME upon EZH2 inhibition as well as functional alterations of the Treg population resulting in Treg-mediated pro-inflammatory activities [93, 103, 121, 122]. Additionally, targeting EZH2 has been reported as an attractive strategy to combine with immunotherapy, as it might overcome resistance to immune checkpoint therapies, including CTLA-4, PD-1 and PD-L1 [93, 121–123]. Among the EZH2-inhibiting compounds, small molecule inhibitors of EZH2, GSK-126, PF-06821497, MAK683, CPI-0209, CPI-1205 and DS-3201 have entered into clinical trials, although none of these has been approved for cancer treatment, yet [122]. Tazemetostat, a selective inhibitor of EZH2, has been approved by the Food and Drug Administration (FDA) in 2020.

Another relevant aspect is the profound interaction between the different immune cell populations in immune response regulation. Therefore, it is important to take into account that epigenetic reprogramming of a certain immunosuppressive immune cell population might positively co-opt other immune cell populations to provoke an effective anti-tumor immune response. One example is the Treg-mediated pro-inflammatory function upon EZH2 inhibition causing increased effector-T cell infiltration and activity. Furthermore, MDSCs play a central role in the immunosuppressive, tumor-promoting TME and have been found to interact with many of the other immune cell populations. Accordingly, epigenetic targeting of this cell type might be sufficient to modify the TME to become more “receptive” to the development of an effective anti-tumor response.

## Immunotherapy—an overview

The immune system plays a critical role in cancer development and progression by both eliminating cancer cells and determining tumor immunogenicity [124]. Thus, cancer immunoediting helps to understand how tumors escape the immune system by dividing the process in three distinct phases: “elimination,” “equilibrium” and “escape.” At first, when cancer cells are present, the immune system can recognize these and eliminate them. However, when not all cancer cells are eradicated in this process and an equilibrium is reached, the adaptive immune system impedes tumor’s growth associated with a dormancy state and high genomic instability. T cells, IL-12 and interferon (IFN)-γ are needed to maintain tumor dormancy [125, 126]. Subsequently, cancer cells escape from the immune system by expressing suppressive effects and losing target antigen expression. At this stage, tumor immune escape occurs, since the adaptive immune system fails to recognize cancer cells, which became resistant to immune effector mechanisms and induced an immunosuppressive state [127].

Immune responses are regulated by an interplay of costimulatory and inhibitory signals that balance the immune response and self-tolerance [128]. Immune checkpoint inhibitors are essential as negative signals to stop immune response and impede autoimmunity [129]. PD-1 is expressed in T lymphocytes and prevents the activation of these cells by binding to its ligands PD-L1 and PD-L2 [130]. Additionally, cytotoxic T lymphocyte-associated antigen 4 (CTLA-4) leads to suppression of T-cells activation by competing with the costimulatory signal CD28 for binding to B7-1 and B7-2, attenuating the activation signals of CD28 [131, 132]. Interestingly, PD-L1 is overexpressed in cancer cells, facilitating cancer cells to escape immune surveillance by T cells [133].

Checkpoint inhibitor immunotherapies consist of monoclonal antibodies that target CTLA-4 or the programmed cell death protein 1 pathway (PD-L1, PD-1) [133]. When the antibodies bind to PD-L1/PD-1 or CTLA-4, the inhibitory effect is canceled and an immunological response against cancer cells starts by activation of tumor-reactive T cells [134]. Of note, several clinical trials have demonstrated increased efficacy of combining anti PD-L1/PD1 and anti-CTLA4, although with increased risk of adverse reactions. The use of immune checkpoint inhibitors as cancer therapy was firstly approved for treatment of metastatic melanoma. Since then, several antibodies have been approved for treatment of non-small cell lung cancer, head and neck squamous cell carcinoma, hepatocellular carcinoma and BC [134].

A significant subset of cancer patients does not respond or respond poorly to immune checkpoint blockage treatments [135]. This can be a consequence of primary resistance that occurs prior to treatment, associated with a reduction of antigen expression and changes in metabolic pathways or through acquired resistance during the course of the treatment [136]. The one and foremost biomarker used for prediction of response to PD-L1/PD-1 blockade is PD-L1 expression [137]. It seems to be a biomarker of aggressive disease and it might also be considered a prognostic biomarker. However, the evaluation of PD-L1 as a single biomarker across clinical trials was shown to be heterogeneous [138]. Several reasons can be appointed for the presence of this heterogeneity: (1) clinical trials used different PD-L1 immunohistochemistry scoring assays [137]; (2) the scoring compartment differs for each specific therapy, namely pembrolizumab and nivolumab use PD-L1 tumor cell expression, whereas atezolizumab uses PD-L1 immune cell expression; (3) intratumoral heterogeneity; and (4) the sample available may not represent the full intratumoral heterogeneity [139]. All these data indicate that PD-L1 expression as a single biomarker is probably not adequate to accurately predict immunotherapy response and more reliable biomarkers could help to better predict and improve patient selection for these therapies [139].

Other approaches have been used to try to predict immunotherapy response including tumor mutational burden, tumor mismatch-repair deficiency, grade of TILs [140–142], among others, depending on tumor type. For example, the TCGA-based molecular subtypes in BC have been associated with response to immune checkpoint blockage. Specifically, the neuronal subtype seems to display a better response to immunotherapy [143]. However, the criteria to define BC subtypes have to be uniformed before it may be considered a possible biomarker for immunotherapy [144]. Moreover, tumor mutational burden has been proposed to predict response to immunotherapy [145]. With a high rate of mutations, novel antigens emerge regularly so it could potentiate the use of immunotherapy. Nonetheless, some patients with low mutational burden endure response to immunotherapy, which demonstrates that criteria to define the tumor mutational burden have to be clarified [145]. Interestingly, pembrolizumab was approved for patients with microsatellite instability and mismatch repair-deficient malignancies showing progression after failure of other approved treatments [142]. Finally, immune expression profiling has the potential to correctly identify “hot” or “cold” tumors by assessing levels of chemokines, cytokines and cell surface proteins reflecting the inflammatory status [146]. Also, it can take into account the several cell types present in the TME, which can be useful in defining immunotherapy response [147]. Examples include IFN-γ, CXCL9 and CXLC10 whose expression correlates with response to immunotherapy [146, 148].

Epigenetic mechanisms are known to regulate several aspects related to immune regulation and actions [149]. 5-Azacytidine (5-aza), a demethylating agent, was shown to upregulate innate and adaptative immune-related genes, specifically to immune invasion, such as PD-L1 at both transcript and protein levels without altering CD80 and CD86. Furthermore, genes related to antigen presentation including HLA class I, *B2M*, *CD58*, *TAP1*, *PMSB9* and *PSMB8* were upregulated after 5-aza treatment [150]. Treatment of leukemia cells with decitabine (DAC) translated into upregulation of PD-L1, PD-L2, PD-1 and CTLA-4 in these cells [151]. This topic is thoroughly discussed in a recently published review [152]. A DNA methylation-based profile—EPIMMUNE signature—of stage IV non-small cell lung cancer patients treated with anti-PD-1 therapies associated with improved progression-free and overall survival. EPIMMUNE-negative tumors disclosed a TME enriched in TAMs, cancer-associated fibroblasts and neutrophils. Moreover, unmethylated *FOXP1* associated with better progression-free and overall survival [153]. Promoter methylation of *RAD51B* seems to associate with PD-L1 expression in lung cancer patients, with high levels of *RAD51B* methylation associating with lower risk of disease progression. Remarkably, combining *RAD51B* methylation and PD-L1 improved sensitivity to predict response to anti-PD-1 blockade and associated with a lower risk of death [154].

Although several studies showed that modulating epigenetic marks can improve therapeutic response to immune checkpoint inhibitors, the search for biomarkers is ongoing and needs further exploitation (Additional file 1: Table 1).

## Epidrugs in cancer

Epigenetic mechanisms including DNA methylation and histone post-translational modifications represent an alluring target for cancer therapy since they are reversible alterations important for tumor cells’ development [155, 156]. Therefore, a class of compounds that came to be known as “epidrugs” were developed targeting these alterations (Table 2) [157]. Currently, DNMT and HDAC inhibitors are the most used epidrugs for cancer treatment in both clinic and clinical trials, namely in combination with chemo- and immunotherapy (Table 2).

**Table 2:** Epidrugs approved and in clinical trial for cancer treatment, with respective effects and drug category

Drug	Approved	Clinical trial	Effects
Azacitidine Vidaza®	High-risk myelodysplastic syndromes Chronic Myelomonocytic Leukemia Acute Myeloid Leukemia	Pancreatic, colorectal, prostate, esophageal, breast, non-small cell lung cancer, thyroid, ovarian, nasopharyngeal and bladder cancer, hematological malignancies sarcoma, melanoma, germ cell tumors and renal cell carcinomas	Nucleoside DNMT inhibitor
Decitabine Dacogen®	High-risk myelodysplastic syndromes Chronic Myelomonocytic Leukemia Acute Myeloid Leukemia	Ovary, head and neck, colorectal, breast, esophageal, non-small cell lung, prostate, thyroid cancers, B cell lymphoma, glioma and medulloblastoma	Nucleoside DNMT inhibitor
5′-Fluoro-2′-deoxycytidine (FdCyd)	–	Head and neck, lung, urinary bladder, breast cancer and acute myeloid leukemia	Nucleoside DNMT inhibitor
Guadecitabine	–	Kidney, lung, ovarian, prostate, colorectal, gallbladder, pancreatic, urothelial cancer, extrahepatic bile duct adenocarcinoma, biliary type, testicular germ cell tumors, chondrosarcoma, melanoma, acute myeloid leukemia and myelodysplastic syndrome	Nucleoside DNMT inhibitor
RX-3117 (fluorocyclopentenylcytosine)	–	Pancreatic and bladder cancer	Nucleoside DNMT inhibitor
Genistein	–	Breast, prostate, colorectal, lung, pancreatic, bladder, kidney, endometrial cancer and melanoma	Isoflavone non-nucleoside DNMT inhibitor
Curcumin	–	Prostate, colorectal, breast, lung, head and neck and cervical cancer	Natural phenol non-nucleoside DNMT inhibitor
Hydralazine	–	Ovarian, cervical, lung and breast cancer	Repurposed drug non-nucleoside DNMT inhibitor
Belinostat Beleodaq®	Peripheral T-cell Lymphoma	Lung, breast, ovary, hematological malignancies, bladder, liver cancer and chondrosarcoma	Hydroxamic acid pan-HDAC inhibitor
Givinostat	–	Chronic Myeloproliferative Neoplasms Polycythemia Vera	Hydroxamic acid pan-HDAC inhibitor
Panobinostat Farydak®	Multiple Myeloma	Breast, lung, pancreatic, prostate, colorectal, head and neck, esophageal, neuroendocrine, renal, thyroid, brain cancer, hematologic neoplasms and melanoma	Hydroxamic acid pan-HDAC inhibitor
Trichostatin A	–	Hematological malignancies	Hydroxamic acid pan-HDAC inhibitor
Vorinostat Zolinza®	Cutaneous T cell Lymphoma	Breast, ovarian, pancreatic, lung, colorectal, gastric, liver, prostate, renal, bladder brain cancer, melanoma and hematological malignancies	Hydroxamic acid pan-HDAC inhibitor
Entinostat	–	Breast, colorectal, ovarian, neuroendocrine, lung, prostate, renal, pancreatic, endometrial cancer, hematological malignancies and cholangiocarcinoma	Benzamide class I HDAC inhibitor
Romidepsin Istodax®	Cutaneous T-cell lymphoma	Lung, breast, pancreatic, colorectal, thyroid, bladder, ovarian cancer, glioma and hematological malignancies	Benzamide class I HDAC inhibitor
Valproic acid	–	Cervical, brain, lung. breast, pancreatic, prostate, bladder, thyroid, head and neck cancer and hematological malignancies	Short-chain and aromatic fatty acids pan-HDAC inhibitor
Abexinostat	–	Breast cancer, renal cell carcinoma, sarcoma, melanoma and hematologic malignancies	Hydroxamic acid pan-HDAC inhibitor
Tazemetostat Tazverik®	Advanced epithelioid sarcoma Follicular lymphoma	Prostate, ovarian, endometrial, head and neck cancer, melanoma, hematological malignancies, urothelial carcinoma and malignant mesothelioma	EZH2 inhibitor
Domatinostat	–	Melanoma and Merkel cell carcinoma	Benzamide class I HDAC inhibitor

DNMT inhibitors are classified into two main groups: nucleoside and non-nucleoside analogues (Table 2) [158]. 5-aza was the first compound developed and approved for clinical usage for DNMT inhibition. Indeed, 5-aza and decitabine (5-aza-2′-deoxycytidine) are already approved for treatment of hematological malignancies including acute myeloid leukemia (AML), myelodysplastic syndrome and chronic myelomonocytic leukemia (CMML) by European Medicines Agency (EMA) and FDA (Table 3) [157, 159, 160]. Nucleoside analogues include analogues of cytosine that are integrated in DNA and lead to the formation of a covalent bond with the DNMT, which results in its degradation and DNA methylation inhibition [157, 161]*.* Both are nucleoside analogues characterized by replacing cytosine during DNA replication, which translates in the formation of a covalent bond when DNMTs exert their function, resulting in DNMTs’ inhibition and further degradation [161–163]. Despite both showing to have anti-tumoral effects, namely by inducing apoptosis, cell and re-expression of tumor suppressor genes [164, 165], 5-aza and DAC display low bioavailability and a limited half-life, which hinders their wide implementation in clinical practice [161, 166]. More stable compounds with the same effect and mechanism of action have been described in the past years including zebularine, 5′-fluoro-2′-deoxycytidine (FdCyd), guadecitabine and RX-3117. For example, FdCyd, a fluoropyrimidine analogue, is less toxic and more stable when compared to 5-aza and DAC (77). Furthermore, FdCyd showed to stop cell arrest at G2/M in HCT116 cells though the DNA damage response pathway [167]. RX-3117 (fluorocyclopentenylcytosine) is a recent cytidine analogue with a modified ribose molecule being activated specifically by uridine-cytidine kinase 2 (UCK2), leading to DNA damage and lower DNA methylation levels though DNMT1 inhibition [168]. Another compound that shows great stability, including being possible to administer orally, is zebularine [169], which showed to have an specific effect on cancer cells and not fibroblasts [170, 171]. However, since it showed great toxicity in primates, it was not continued for clinical trials (Table 3) [172, 173]. Guadecitabine is a CpG dinucleotide analogue hypomethylating agent [174] with proven anti-tumoral activity, such as in combination with cisplatin in platinum-refractory germ cell tumors [175].

**Table 3:** Major clinical trials comprising epidrugs and immunotherapy for cancer treatment

Clinical trial	Phase	State	Cancer	Combination	Dates
NCT01928576	II	Recruiting	Non-small cell lung cancer	Nivolumab Entinostat Azacitidine	2013-estimated end 2022
NCT02260440	II	Completed	Metastatic colorectal cancer	Pembrolizumab Azacitidine	2015–2017
NCT02546986	II	Active, not recruiting	Non-small cell lung carcinoma	CC-486 Pembrolizumab	2015-estimated end 2021
NCT02453620	I	Active, not recruiting	Breast Cancer	Ipilimumab Nivolumab Entinostat	2015-estimated end 2021
NCT02961101	I/II	Recruiting	Non-Hodgkin lymphoma, Hodgkin lymphoma, gastrointestinal cancers, hepatocellular carcinoma, breast cancer, ovarian cancer, lung cancer, renal-cell cancer, pancreatic cancer and bile duct cancer	Anti-PD-1 antibody Decitabine Chemotherapy	2016–2020
NCT02890069	I	Recruiting	Colorectal cancer Non-small cell lung adenocarcinoma Triple-negative breast cancer Renal cell carcinoma	PDR001 (Anti-PD-1 antibody) Everolimus Panobinostat	2016-estimated end 2021
NCT02619253	I/Ib	Active, not recruiting	Renal cell carcinoma Urinary bladder neoplasms	Pembrolizumab Vorinostat	2016-estimated end 2022
NCT02512172	I	Active, not recruiting	Colorectal cancer	CC-486 (Oral 5-Aza) Romidepsin MK–3475 (Anti-PD-1 antibody)	2016-estimated end 2022
NCT02811497	II	Active, not recruiting	Microsatellite stable colorectal carcinoma Platinum-resistant epithelial ovarian cancer type II Estrogen receptor-positive and HER2-negative breast cancer	Durvalumab Azacitidine	2016-estimated end 2022
NCT02957968	II	Recruiting	Breast cancer	Decitabine Pembrolizumab Doxorubicin Cyclophosphamide Paclitaxel Carboplatin	2016-estimated end 2023
NCT02638090	I/II	Recruiting	Lung cancer	Vorinostat Pembrolizumab	2016-estimated end 2023
NCT02959437	I/II	Completed	Advanced or metastatic solid tumors	Azacitidine Pembrolizumab Epacadostat INCB057643 (BET inhibitor) INCB059872 (LSD1 inhibitor)	2017–2020
NCT03308396	Ib/II	Active, not recruiting	Advanced kidney cancer Kidney cancer Clear cell renal cell carcinoma	Durvalumab Guadecitabine	2017-estimated end 2021
NCT03206047	I/II	Active, not recruiting	Platinum-resistant fallopian tube carcinoma Platinum-resistant ovarian carcinoma Platinum-resistant primary peritoneal carcinoma Recurrent fallopian tube carcinoma Recurrent ovarian carcinoma Recurrent primary peritoneal carcinoma	Atezolizumab Guadecitabine	2017-estimated end 2021
NCT03264404	II	Recruiting	Pancreas cancer	Pembrolizumab Azacitidine	2017-estimated end 2021
NCT03179943	II	Active, not recruiting	Urothelial carcinoma	Atezolizumab Guadecitabine	2017-estimated end 2022
NCT03019003	I/II	Recruiting	Head and neck cancer	Durvalumab Oral Decitabine	2017-estimated end 2024
NCT02816021	II	Recruiting	Melanoma and other malignant neoplasms of skin Metastatic melanoma	Pembrolizumab Azacitidine	2017-estimated end 2026
NCT03590054	I	Recruiting	Melanoma Metastatic head and neck squamous cell carcinoma Urothelial carcinoma Non-small cell lung carcinoma	Pembrolizumab Abexinostat	2018-estimated end 2022
NCT03426891	I	Recruiting	Glioblastoma Brain tumor	Pembrolizumab Vorinostat Temozolomide	2018-estimated end 2022
NCT03233724	I/II	Recruiting	Non-small cell lung Cancer Esophageal carcinoma Malignant pleural mesotheliomas	Pembrolizumab Decitabine Tetrahydrouridine (THU)	2018-estimated end 2026
NCT03854474	I/II	Recruiting	Locally and metastatic urothelial carcinoma	Pembrolizumab Tazemetostat	2019-estimated end 2021
NCT03812796	II	Recruiting	Gastrointestinal cancer	Domatinostat Avelumab	2019-estimated end 2021
NCT03765229	II	Recruiting	Melanoma	Pembrolizumab Entinostat	2019-estimated end 2023
NCT03978624	II	Recruiting	Bladder cancer	Pembrolizumab Entinostat	2020-estimated end 2022
NCT04357873	II	Recruiting	Squamous cell lung cancer Vulvar cancer Penile cancer Cervix cancer Head and neck squamous cell carcinoma Anal cancer	Pembrolizumab Vorinostat	2020-estimated end 2024
NCT04624113	I/II	Not yet recruiting	Head and neck squamous cell carcinoma	Pembrolizumab Tazemetostat	2021-estimated end 2024
NCT04190056	II	Not yet recruiting	Breast cancer	Pembrolizumab Tamoxifen Vorinostat	2021-estimated end 2029

Another heterogeneous group of compounds with DNMT inhibition activity is the non-nucleoside analogs, which inhibit DNMTs independently from DNA incorporation (Table 2) [176]. These include natural compounds, DNA binders, SAM competitors and repurposed drugs [176, 177]. Regarding natural compounds, molecules such as genistein, the natural polyphenol epigallocatechin-3-gallate (EGCG) and curcumin that displays anti-inflammatory and anti-oxidant properties showed to inhibit DNA methylation, translating in anti-tumoral activity [158, 178–180]. 3-Halo-3-nitroflavanones, a novel class of DNMT inhibitors described recently, showed anti-tumoral activity associated with higher stability and low cytotoxicity than the most used DNMT inhibitors [181]. Interestingly, a compound from this family MLo1302 caused a decrease in cell viability, namely in cisplatin-resistant cell lines, pluripotency markers and an activation of apoptosis and cell cycle arrest in germ cell tumors cell lines [182]. RG108 is a SAM competitor that binds to the catalytic pockets of DNMTs, forming covalent bonds and inhibiting the enzyme action [183]. Treatment with RG108 caused radiosensitivity of esophageal cancer cells, enhancing apoptosis and G2/M phase arrest by radiation [184]. SGI-1027, a quinolone-based molecule which binds to both DNMT3a and DNMT1 [185]*,* combined with AH057 (JAK inhibitor) increased cervical cancer cells apoptotic cell death and cell-cycle arrest [186]. Furthermore, MG98 is a second-generation 20-nucleotide antisense oligonucleotide that binds specifically to DNMT1 mRNA, decreasing DNMT1 levels [187].

Selected repurposed drugs, designed for a specific treatment that were found to have other therapeutic targets, also showed effect against DNMTs [176, 177]*.* For example, procaine and procainamide, approved as anesthetic and anti-arrhythmic drugs, respectively, bind to the catalytic center of DNMTs, which translated in a decrease in DNA methylation levels [188, 189]. The arterial vasodilator hydralazine also leads to the loss of promoter hypermethylation of TSGs in cancer cell lines and primary tumors [190, 191]. In this line, the antibiotic nanaomycin A also causes the same effects, being selective for DNMT3b [192].

HDAC inhibitors can be classified according to chemical group as hydroxamic acids, short-chain and aromatic fatty acids, benzamides and cyclic peptides (Table 2). The hydroxamic acids and cycle peptides constitute the most potent inhibitors, with IC_50_ in the low micro- or nanomolar range, while short-chain fatty acids require doses in the millimolar range [193].

Hydroxamic acids include belinostat (approved for peripheral T-cell lymphoma), givinostat, panobinostat, trichostatin A and vorinostat, all pan-HDAC inhibitors. Vorinostat or suberanilohydroxamic acid (SAHA) was the first HDAC inhibitor to be approved for the treatment of advanced cutaneous T-cell lymphoma, and it shows several anti-tumor effects in several hematological and solid tumors in vitro and in vivo (Table 3) [194]. It inhibits class I and II HDACs by binding to the catalytic domain of the enzymes [195]. Similar to vorinostat, trichostatin A (TSA), also a class I and II inhibitor, acts as a non-competitive inhibitor of HDAC by mimicking the lysin substrate as a chelating agent to the zinc atom in the active site [195]. Interestingly, it showed to reverse chemoresistance in lung cancer cell lines with high expression of IGFBP2, with IGFBP2 being a biomarker of chemoresistance poor outcome in lung cancer patients [196]. Panobinostat is approved for patients with recurrent multiple myeloma who have received at least two prior treatment regiments (Table 3) [193]. Indeed, the progression-free survival of patients treated with panobinostat was 10.6 months in comparison with 5.8 months in the control arm [197]. Belinostat also inhibits HDACs by binding to the zinc finger in the enzymes’ active catalytic site and, recently, showed to be active in testicular germ cell tumor cell lines resistant to cisplatin, with IC_50_ in the low nanomolar range for all cell lines [198].

Entinostat belongs to the benzamide group of HDAC inhibitors and inhibits class I HDACs [193]. Remarkably, entinostat boosted the effects of PD-1 inhibition in in vivo models of lung and renal cancers by impairing the immunosuppressive function of polymorphonuclear and monocytic-myeloid derived suppressor cell populations [112]. Another inhibitor of class I HDACs is romidepsin (FK228) belonging to the cyclic peptide family [193]. Romidepsin has been approved by the FDA for the treatment of advanced cutaneous T-cell lymphoma and peripheral T-cell lymphoma (Table 3) [199]. The triple combination of romidepsin, gemcitabine and cisplatin acted synergically and induced death by creating reactive oxygen species in triple-negative breast cancer cell lines [200].

The short-chain and aromatic fatty acids sodium butyrate and valproic acid also were shown to be pan-HDACs inhibitors [193]. Sodium butyrate is a short-chain fatty acid produced by fermentation by anaerobic bacterial fermentation that inhibits growth, induces apoptosis, migration and EMT of colorectal cancer cells [201]. Valproic acid, used for the treatment of epilepsy and bipolar disorder, in combination with cisplatin and cetuximab, showed antiproliferative and pro-apoptotic effects in 3D-self-assembled spheroid models of ad and neck squamous cell carcinoma cells [202].

## Bladder cancer

BC is estimated to be the tenth most frequent cancer worldwide and the ninth cause of death by cancer [203]. About 70% of the patients are diagnosed as a non-muscle invasive BC (NMIBC), while 30% are diagnosed with muscle invasive BC (MIBC) [204]. NMIBC is mostly comprised of urothelial papillary neoplasms with varying propensity for recurrence and progression, which may be predicted based on grading [205]. According to the 2016 WHO classification, the spectrum of papillary neoplasms includes urothelial papilloma, papillary urothelial neoplasm of low malignant potential, low-grade and high-grade papillary urothelial carcinoma, in ascending order of biological and clinical aggressiveness [206]. On the other hand, urothelial carcinoma in situ (CIS) represents a high-grade form of non-papillary NMIBC, with substantial risk of progression to invasive disease [206]. Although NMIBC mostly contributes to the overall BC 5-year survival rate of 77.1%, 80% of high-grade papillary carcinomas and CIS recur and 20–50% progress to MIBC [207]. In addition to grade, evaluation of disease stage [by means of clinical examination, cystoscopy, radiographic evaluation and/or pathological examination using tissue collected by transurethral resection of the bladder tumor (TURBT)] is mandatory to define the best therapeutic strategy [208, 209]. For NMIBC, treatment mostly consists of TURBT eventually complemented with mitomycin or Bacillus Calmette-Guérin (BCG) instillation, whereas radical cystectomy with lymphadenectomy remains the gold standard for MIBC, complemented with neo-adjuvant or adjuvant cisplatin-based chemotherapy, which is also the main option for metastatic BC [209]. Recently, immunotherapies targeting PD-1/PD-L1 immune checkpoint were approved for BC patients that are refractory or ineligible to cisplatin-based chemotherapy [210]. Although chemotherapy and immunotherapy have improved the outcome of locally advanced and metastatic disease, 5-year survival remains poor (36% and 5%, respectively) [207].

**Fig. 3:**
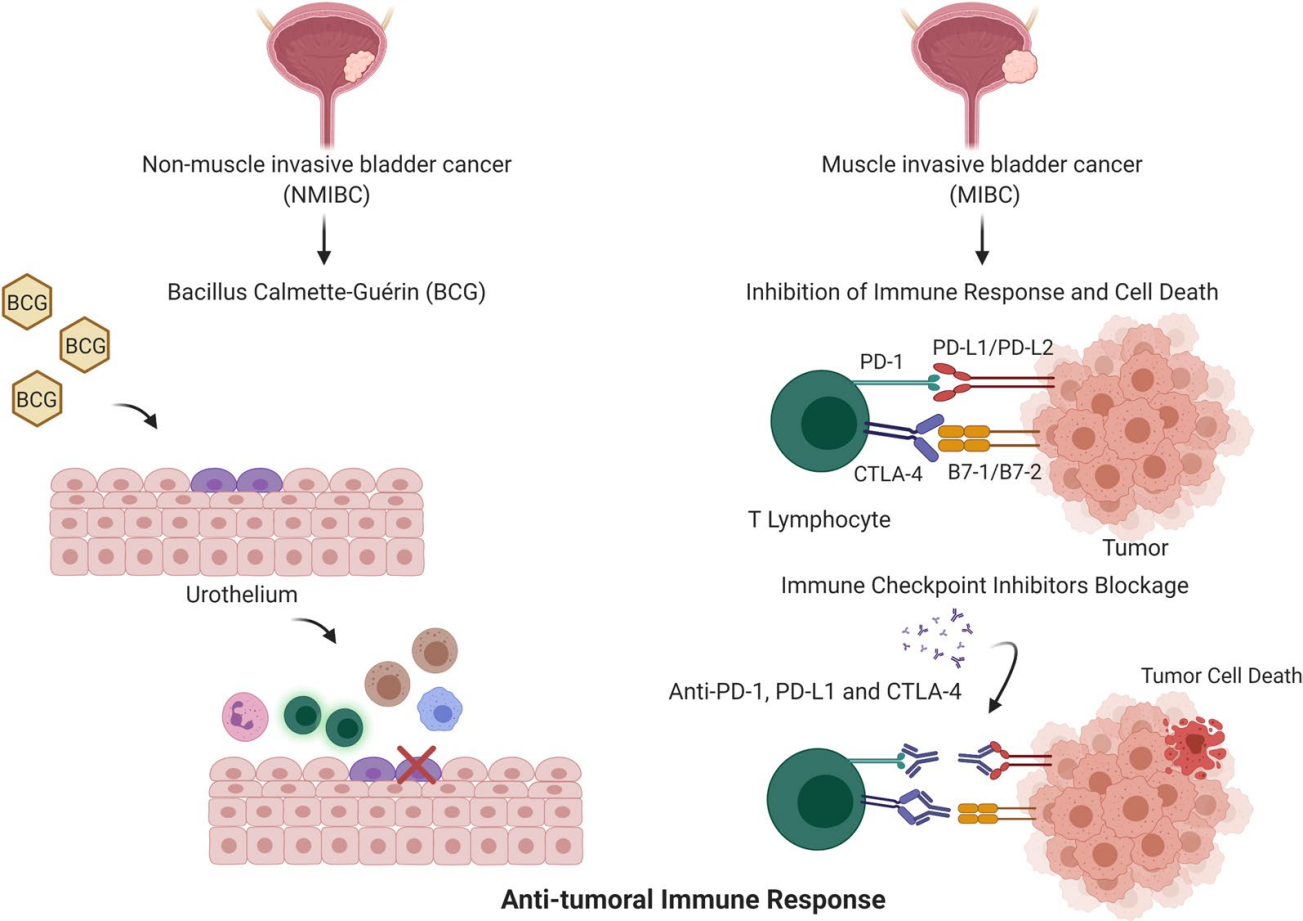
Modalities of immunotherapy in bladder cancer. BCG is a weakened strain of *Mycobacterium bovis* and was the first of immunotherapy approved for BC. NMIBC patients with high risk of recurrence are subjected to BCG therapy. The administration of BCG leads to a stimulation of both adaptative and innate immune response by recruiting lymphocytes, macrophages, NK cells and neutrophils, leading to the elimination of the remaining tumor cells. On the other hand, BC patients with MIBC are candidates for immune checkpoint blockage. Tumor cells express repression signals that lead to the inhibition of the immune response, namely by expressing PD-L1/PD-L2 and B7-1/B7-2, that will bind to PD-1 and CTLA-4 present in T lymphocytes, respectively. Nevertheless, with the administration of antibodies against PD-1, PD-L1 or CTL4-A, this process is reverted, leading to the activation of T cells and the start of an immune response against tumor cells, leading ultimately to their death

BCG, which is a weakened strain of *Mycobacterium bovis*, was the first form of immunotherapy approved for cancer treatment and, specifically, for BC. Currently, it is administrated by intravesical instillation after TURBT in NMIBC patients with high risk of recurrence [211]. Although the mechanism is not fully known, BCG leads to localized innate and adaptative immune responses, including CD4 and CD8 lymphocytes, NK cells, macrophages, granulocytes and DCs [212]. About 55–75% of the high-risk patients suffering from papillary tumors to CIS respond to this therapy. However, 25 to 45% of these eventually relapse and progress to invasive disease. Hypermethylation of *CDKN2B* and of *MUS81a* and *MSH6* involved in DNA repair and *THBS1*, important for cell adhesion, have been associated with response to BCG therapy [210, 213]. Likewise, low methylation levels of *PMF1* have been associated with disease recurrence, poor outcome and lack of response to BCG in BC patients (Fig. 3) [214].

The immune landscape of BC is composed by different immune populations, including CD8 + T lymphocytes and Th1 CD4 + T lymphocytes. Interestingly, tumor-infiltrating CD4 + lymphocytes were found to be hypomethylated in four lineage loci compared to CD4 + lymphocytes in lymph nodes and blood. Patients with complete response to neoadjuvant chemotherapy (NACT) showed hypomethylation in CD4 + T cells, namely in IFN- γ. Furthermore, shifts in methylation patterns of Th1 CD4 + T cells after NACT show a relocation of cells from blood to the tumor (Fig. 3) [215]. Tissue-resident memory T cells showed low PRF1 DNA methylation levels concomitantly with increased perforin expression [216]. The analysis of DNA methylation in neutrophils and lymphocytes predicted the outcome of BC patients, *i.e.*, high levels of DNA methylation-derived neutrophil-to-lymphocyte ratio associated with poor outcome [217].

Demethylating agents lead to reactivation of TSGs, inhibition of cancer cells’ proliferation and migration, increased apoptosis and activation of IFN pathway in BC [218]. Ramakrishnan et al. showed that low concentrations of DAC lead to the activation of NOTCH1, which may prevent epithelial-mesenchymal transition of tumor cells, thus impairing cancer cell dissemination [219, 220]. Moreover, increased IL-6 levels were observed in DAC-treated cells, and reduction of cytokeratin 5 expression associated with cell differentiation and impaired BC progression [221]. Another epigenetic inhibitor for G9a, CM-272, in combination with cisplatin caused an increase in expression of genes associated with immune response, such as TNF-α, IFN-α and IFN-γ, which correlated with an endogenous retrovirus response. Furthermore, an extensive infiltration of CD8 + T cells and NK cells was observed in tumors and metastases in an in vivo immunocompetent model of MIBC. This was also observed with CM-272 in combination with an anti-PD-L1 antibody, with immune infiltration by CD3 + , CD8 + and NK cells, and the absence of CD4 + and CD163 + cells [222].

## Conclusions

Epigenetic alterations in cells of the TME play a major role in creating an immunosuppressive environment ideal for tumor development, which translates in a lack of effectiveness of immune checkpoint blockage therapies. The inhibition of epigenetic modulators might be an interesting therapeutic option to modify the immunosuppressive TME, and their potential in combination with immunotherapy has already been discussed. Additionally, refining patient selection for immunotherapy by exploring new biomarkers with higher sensitivity and specificity might improve the success rate of this therapy. Combining these findings, exploring aberrant epigenetic marks in both cancer cells and in cells of the TME might provide potential biomarkers for this purpose.

Thus, further studies are needed to increase our knowledge on the epigenetic mechanisms underlying the acquisition of immunosuppressive immune cell phenotypes and how these affect immunotherapy response. Additionally, a promising strategy to generate an immune-promoting TME might be the combination of epigenetic modulator targeting and immunotherapy.

## Supplementary Information

**Additional file 1: Table 1.** Main studies comprising epigenetic drugs and biomarkers associated with immunotherapy.

## Data Availability

Not applicable.

## Abbreviations

5-aza: 5-Azacytidine
FdCyd: 5′-fluoro-2′-deoxycytidine
ALOX15: Arachidonate 15-lipoxygenase
AML: Acute myeloid leukemia
APCs: Antigen-presenting cells
APM: Antigen processing and presentation machinery
Arg1: Arginase 1
BC: Bladder cancer
BCG: Bacillus Calmette-Guérin
CAFs: Cancer-associated fibroblasts
CIS: Carcinoma in situ
CMML: Chronic myelomonocytic leukemia
CTLs: Cytotoxic T cells
CTLA-4: Cytotoxic T-lymphocyte-associated protein 4
DAC: Decitabine
DCs: Dendritic cells
DNMTs: DNA methyltransferases
ECM: Extracellular matrix
EGCG: Epigallocatechin-3-gallate
EMA: European Medicines Agency
EZH2: Enhancer of zeste homolog 2
FDA: Food and Drug Administration
HATs: Histone acetyltransferases
HDACs: Histone deacetylases
IFN: Interferon
JMJD3: Jumonji domain-containing protein D3
KLF4: Kruppel-like factor 4
MDSCs: Myeloid-derived suppressor cells
MIBC: Muscle invasive BC
NACT: Neoadjuvant chemotherapy
NK: Natural killer
NMIBC: Non-muscle invasive BC
PD-1: Programmed death 1
PD-L1: Programmed death-ligand 1
PPARγ: Peroxisome proliferator-activated receptor γ
PRMT1: Protein arginine methyltransferase 1
PTMs: Post-translational modifications
Rb: Retinoblastoma gene
SATB1: Special AT-rich sequence binding 1
SAHA: Suberanilohydroxamic acid
SMYD3: SET and MYND Domain 3
STAT3: Signal transducer and activator of transcription 3
TAAs: Tumor-associated antigens
TAMs: Tumor-associated macrophages
TECs: Tumor endothelial cells
Th: T helper
TILs: Tumor-infiltrating lymphocytes
TME: Tumor microenvironment
Tregs: Regulatory T cells
TSA: Trichostatin A
TSGs: Tumor suppressor genes
TURBT: Transurethral resection of the bladder
UCK2: Uridine-cytidine kinase 2
