# Supplementary material for: Tackling tumor microenvironment through epigenetic tools to improve cancer immunotherapy
Source: Clin Epigenetics. 2021 Mar 24;13:63. doi: 10.1186/s13148-021-01046-0 (PMC7992805; doi:10.1186/s13148-021-01046-0)
Supplement: Supplementary file 1 — Additional file 1: Table 1. Main studies comprising epigenetic drugs and biomarkers associated with immunotherapy. [file 13148_2021_1046_MOESM1_ESM.docx]

| **Study** | **Major findings** | **Reference** |
| --- | --- | --- |
| Wrangle et al. (2013) | AZA upregulates genes and pathways related to both innate and adaptive immunity and genes related to immune evasion in a several non-small cell lung cancer lines. | [1] |
| Yang et al. (2014) | In a cohort of patients treated with epigenetic therapy, PD-L1, PD-L2, PD-1 and CTLA4 expression was upregulated. Exposure to decitabine resulted in partial demethylation of PD-1 in leukemia cell lines and human samples | [2] |
| Kim et al. (2014) | Entinostat reduces the MDSC population and the combination of entinostat with PD-1 and CTLA-4 antibodies resulted in an elimination of approximately 80% of the tumor, whereas the use of immune checkpoint inhibitors against PD-1 and CTLA-4 alone did not provide an anti-tumor immune response. | [3] |
| Zingg et al. (2017) | Targeting Ezh2 in melanoma cells reverts loss of immunogenicity and antigen presentation, and synergizes with anti-CTLA-4 and IL-2 immunotherapies | [4] |
| Orillion et al. (2017) | Entinostat increases the antitumor effect of PD-1 targeting by functional inhibition of immunosuppressive MDSCs, which helps the TME to become more receptive to the development of an effective anti-tumor response | [5] |
| Briere et al. (2018) | The class I/IV HDAC inhibitor mocetinostat increases tumor antigen presentation, decreases immune suppressive cell types and augments checkpoint inhibitor therapy with PD-L1 antibody. | [6] |
| Goswami et al. (2018) | Targeting EZH2 expression in T cells (CPI-1205) can improve anti-tumor immune responses provided by immune checkpoint inhibitor CTLA-4 (Ipilimumab). | [7] |
| Duruisseaux et al. (2018) | The methylation status of FOXP1 could be associated with validated predictive biomarkers such as PD-L1 staining and mutational load to better select patients who will experience clinical benefit with PD-1 blockade. | [8] |
| Segovia et al. (2019) | The antitumor effect was significantly improved when CM-272 was combined with anti-programmed cell death ligand 1. These effects were associated with an endogenous antitumor immune response and immunogenic cell death with the conversion of a cold immune tumor into a hot one. | [9] |
| Guerreiro et al. (2020) | RAD51Bme levels might be combined with validated predictive biomarker PD-L1 immunostaining to select patients who will most likely experience clinical benefit from PD-1 blockade | [10] |
| Lai et al. (2021) | Zebularine promoted infiltration of CD8 T cells and natural killer (NK) cells into tumor, decreasing tumor growth. Furthermore, zebularine sensitized the cGAS-STING pathway to promote anti-tumor immunity. | [11] |

**Table 1.** Main studies comprising epigenetic drugs and biomarkers associated with immunotherapy.

**References**

1. Wrangle J, Wang W, Koch A, Easwaran H, Mohammad HP, Vendetti F, et al. Alterations of immune response of non-small cell lung cancer with Azacytidine. Oncotarget [Internet]. Impact Journals LLC; 2013 [cited 2020 Nov 30];4:2067–79. Available from: https://pubmed.ncbi.nlm.nih.gov/24162015/

2. Yang H, Bueso-Ramos C, Dinardo C, Estecio MR, Davanlou M, Geng QR, et al. Expression of PD-L1, PD-L2, PD-1 and CTLA4 in myelodysplastic syndromes is enhanced by treatment with hypomethylating agents. Leukemia [Internet]. Nature Publishing Group; 2014 [cited 2020 Nov 30];28:1280–8. Available from: https://pubmed.ncbi.nlm.nih.gov/24270737/

3. Kim K, Skora AD, Li Z, Liu Q, Tam AJ, Blosser RL, et al. Eradication of metastatic mouse cancers resistant to immune checkpoint blockade by suppression of myeloid-derived cells. Proc Natl Acad Sci U S A [Internet]. National Academy of Sciences; 2014 [cited 2020 Dec 3];111:11774–9. Available from: https://www.pnas.org/content/111/32/11774

4. Zingg D, Arenas-Ramirez N, Sahin D, Rosalia RA, Antunes AT, Haeusel J, et al. The Histone Methyltransferase Ezh2 Controls Mechanisms of Adaptive Resistance to Tumor Immunotherapy. Cell Rep [Internet]. ElsevierCompany.; 2017;20:854–67. Available from: http://dx.doi.org/10.1016/j.celrep.2017.07.007

5. Orillion A, Hashimoto A, Damayanti N, Shen L, Adelaiye-Ogala R, Arisa S, et al. Entinostat neutralizes myeloid-derived suppressor cells and enhances the antitumor effect of PD-1 inhibition in murine models of lung and renal cell carcinoma. Clin Cancer Res [Internet]. American Association for Cancer Research Inc.; 2017 [cited 2020 Oct 19];23:5187–201. Available from: https://pubmed.ncbi.nlm.nih.gov/28698201/

6. Briere D, Sudhakar N, Woods DM, Hallin J, Engstrom LD, Aranda R, et al. The class I/IV HDAC inhibitor mocetinostat increases tumor antigen presentation, decreases immune suppressive cell types and augments checkpoint inhibitor therapy. Cancer Immunol Immunother [Internet]. Springer Science and Business Media Deutschland GmbH; 2018 [cited 2020 Dec 3];67:381–92. Available from: https://pubmed.ncbi.nlm.nih.gov/29124315/

7. Goswami S, Apostolou I, Zhang J, Skepner J, Anandhan S, Zhang X, et al. Modulation of EZH2 expression in T cells improves efficacy of anti-CTLA-4 therapy. J Clin Invest. 2018;128:3813–8.

8. Duruisseaux M, Martínez-Cardús A, Calleja-Cervantes ME, Moran S, Castro de Moura M, Davalos V, et al. Epigenetic prediction of response to anti-PD-1 treatment in non-small-cell lung cancer: a multicentre, retrospective analysis. Lancet Respir Med [Internet]. Lancet Publishing Group; 2018 [cited 2020 Dec 13];6:771–81. Available from: https://pubmed.ncbi.nlm.nih.gov/30100403/

9. Segovia C, San José-Enériz E, Munera-Maravilla E, Martínez-Fernández M, Garate L, Miranda E, et al. Inhibition of a G9a/DNMT network triggers immune-mediated bladder cancer regression. Nat Med [Internet]. Nature Publishing Group; 2019 [cited 2020 Oct 10];25:1073–81. Available from: https://pubmed.ncbi.nlm.nih.gov/31270502/

10. Guerreiro IM, Barros-Silva D, Lopes P, Cantante M, Cunha AL, Lobo J, et al. RAD51Bme Levels as a Potential Predictive Biomarker for PD-1 Blockade Response in Non-Small Cell Lung Cancer. J Clin Med [Internet]. MDPI AG; 2020 [cited 2020 Dec 13];9:1000. Available from: https://pubmed.ncbi.nlm.nih.gov/32252414/

11. Lai J, Fu Y, Tian S, Huang S, Luo X, Lin L, et al. Zebularine elevates STING expression and enhances cGAMP cancer immunotherapy in mice. Mol Ther [Internet]. Elsevier; 2021 [cited 2021 Feb 19];0. Available from: https://linkinghub.elsevier.com/retrieve/pii/S1525001621000721
